# The Diagnosis and Treatment of Diseases of the Ear

**Published:** 1884-10

**Authors:** 


THE DIAGNOSIS AND TREATMENT OF DISEASES OF THE EAR. *By* OREN D. POMEROY, M. D. *Surgeon to the Manhattan Eye and Ear Hospital; Ophthalmic and Aural Surgeon to the N. Y. Infant Asylum; Consulting Surgeon to the Paterson Eye and Ear Infirmary, Etc., Etc. With one hundred illustrations. New York:* BERMINGHAM & CO. *1883.*

In the preface of this book, the author calls our attention to the absence of any reference to the anatomy and physiology of the ear. He refers the student to special works for a thorough study of these subjects. As the book is intended for practitioners and young physicians, we cannot approve of this omission. Physicians, in the active pursuit of their vocation, surely have not the time nor the inclination for exhaustive study, especially of so dry a subject as the anatomy of the ear. Still a certain knowledge of the structure of this organ is highly essential for a thorough understanding of the pathology and treatment of the diseases invading these parts.

We would recommend the author to add to the value of this otherwise commendable text-book, by devoting in the next edition at least several pages to this subject.

Altogether the work is well adapted to the needs of physicians and students. It covers the whole ground of otology in a concise and clear manner. The get-up of the book is quite good. Some of the illustrations, especially those depicting the pathology of the M. T., can be improved upon.

